# Prevalence of smoking and associated factors in people living with HIV undergoing treatment

**DOI:** 10.11606/s1518-8787.2020054001828

**Published:** 2020-11-04

**Authors:** Luciane de Souza Leal Teixeira, Maria das Graças Braga Ceccato, Wânia da Silva Carvalho, Juliana de Oliveira Costa, Palmira de Fátima Bonolo, Jullye Campos Mendes, Micheline Rosa Silveira

**Affiliations:** I Universidade Federal de Minas Gerais Faculdade de Farmácia Belo HorizonteMG Brasil Universidade Federal de Minas Gerais. Faculdade de Farmácia. Programa de Pós-Graduação em Medicamentos e Assistência Farmacêutica. Belo Horizonte, MG, Brasil; II Universidade Federal de Minas Gerais Faculdade de Farmácia Departamento de Farmácia Social Belo HorizonteMG Brasil Universidade Federal de Minas Gerais. Faculdade de Farmácia. Departamento de Farmácia Social. Belo Horizonte, MG, Brasil; III UNSW Sydney Faculty of Medicine Centre for Big Data Research in Health Sydney Australia Centre for Big Data Research in Health. Faculty of Medicine. UNSW Sydney, Sydney, Australia; IV Universidade Federal de Minas Gerais Faculdade de Medicina Departamento de Medicina Preventiva e Social Belo HorizonteMG Brasil Universidade Federal de Minas Gerais. Faculdade de Medicina. Departamento de Medicina Preventiva e Social. Belo Horizonte, MG, Brasil; V Universidade Federal de Minas Gerais Faculdade de Medicina Belo HorizonteMG Brasil Universidade Federal de Minas Gerais. Faculdade de Medicina. Programa de Pós-Graduação em Saúde Pública. Belo Horizonte, st, Brasil

**Keywords:** Long-term survivors of HIV, Risk Factors, Smoking, Alcoholism, Substance-Related Disorders, Cross-Sectional Studies

## Abstract

**OBJECTIVE:**

To estimate the prevalence of smoking and evaluate the factors associated with this outcome in people living with HIV (PLHIV).

**METHODS:**

This is a cross-sectional study of a prospective concurrent cohort of 462 individuals initiating antiretroviral therapy at three HIV/AIDS specialized services in Belo Horizonte between 2015 and 2017. The following smoking status were used: current smoker (CS), former smoker (FS) and non-smoker (NS). Multinomial logistic regression was performed with NS as the reference category.

**RESULTS:**

Most participants were men (81.4%), young (up to 34 years old; 57.2%) and non-white (75.7%). Of the total number of individuals, 27.7% were CS, 22.9% FS, and 49.4% NS. Most smokers were light smokers (65.1%), consumed up to 10 cigarettes per day and had been smoking for more than 10 years (63.3%), starting on average at 17.2 years of age (SD = 5.1). In the multivariate analysis, higher chances of being CS were associated with: being female, having up to 9 years of schooling, current or prior use of alcohol and illicit drugs (marijuana, cocaine and crack) and presenting signs and/or symptoms of anxiety or depression. Higher chances of being FS were associated with having up to 9 years of schooling and current or prior use of alcohol and illicit drugs (marijuana and crack).

**CONCLUSIONS:**

The results show that smoking is highly prevalent among PLHIV, indicating the need for HIV specialized services to prioritize smoking cessation interventions. These interventions should consider the use of alcohol and illicit drugs and be targeted especially to young people, those with low schooling and with signs and/or symptoms of anxiety or depression.

## INTRODUCTION

Smoking is the leading cause of preventable death in the world and, in people living with HIV (PLHIV), it is associated with significant morbidity and mortality^1^. The worldwide prevalence of smoking in PLHIV is estimated between 40% and 70% and, in the general population, it is around 20%^2^^–^^4^.

In Brazil, previous studies have estimated that the prevalence of smoking in PLHIV ranged from 28.9% to 33.6%^5^^–^^7^, whereas this rate is 10.1%^8^ in the general population over 18 years – that is, it is about three times higher in PLHIV. Nevertheless, few studies in developing countries have analyzed the factors associated with smoking in PLHIV.

Smoking is an epidemic health condition that causes physical, psychological, and behavioral dependence, especially attributed to nicotine^9^. The components derived from cigarette burning cause morphofunctional changes of macrophages, B and T lymphocytes (CD4 and CD8) and lymphocins, besides promoting the expression of the HIV-1 gene and, consequently, AIDS^6^^,^^10^^,^^11^. The lower response to the use of antiretroviral therapy (ART) and impaired viral suppression in PLHIV smokers involves the mechanism of the CYP1A1-M1 variant of cytochrome P450 (CYP)^10^.

Both cigarette components and other factors that influence the behavior of PLHIV may be associated with failure of ART in this smoking population. These factors include low adherence to treatment, use of licit and illicit substances, depression and anxiety, and changes in the immune function^10^^,^^7^.

The ART regimens made available by the Unified Health System in Brazil have been recently changed, following international recommendations^9^. In view of this scenario, it is important to understand the associated factors and the prevalence of smoking in PLHIV under treatment to plan interventions that are effectively directed at these individuals.

Our study aims to estimate the prevalence of smoking and factors associated with this outcome, as well as describe the profile of smoking use among PLHIV initiating ART in public reference services in Belo Horizonte (MG).

## METHODS

**Figure 1:**
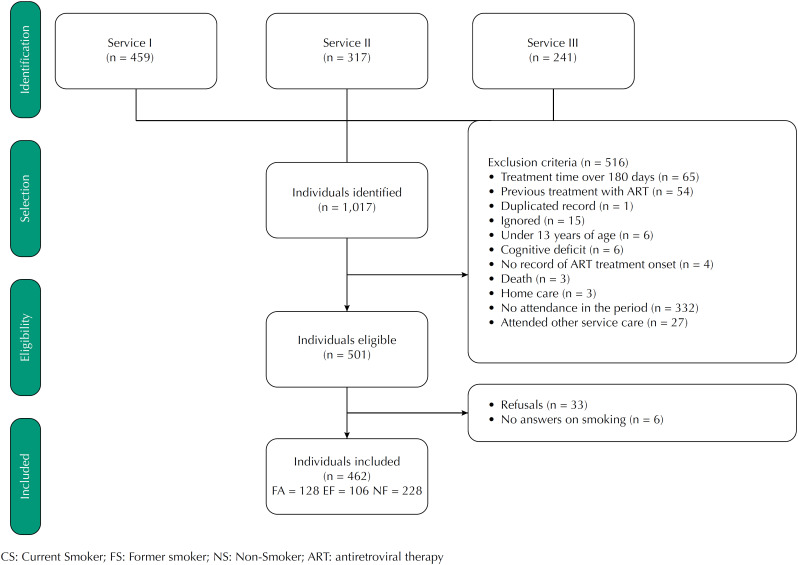
Eligibility diagram of the individuals included in the study. Belo Horizonte, MG, 2017.

This is a cross-sectional study of baseline interviews from the ECOART Project cohort, conducted in Belo Horizonte (MG), Brazil. The selection of the sample was non-random, consisting of 462 individuals in the onset of ART who were identified in the Logistic Drug Control System (Siclom) and in the Laboratory Test Control System of the National Lymphocyte Count Network CD4^+^/CD8^+^ and HIV Viral Load (Siscel). These individuals answered a structured questionnaire applied face-to-face by a trained team (Figure 1).

Recruited occurred from September 2015 to October 2017 in three public services specialized in HIV/AIDS, responsible for dispensing ART for approximately 80% of PLHIV in the city of Belo Horizonte, Minas Gerais. People aged 13 years or older were included. Service I is a HIV specialized service (SAE) that provides inpatient and outpatient care. Service II is a testing and counseling center (CTA), and Service III is an SAE – both linked to the City of Belo Horizonte – which offer interdisciplinary medical care.

The independent variable was smoking cigarettes at the time of the face-to-face interview, measured by the following question: “Do you currently smoke cigarettes?” (yes/no/ignored).

Smokers were classified by the number of cigarettes smoked daily at three levels: light use (up to 10 cigarettes/day), moderate use (10 to 20) or heavy use (above 20), according to the self-report. The number of pack years was calculated by dividing the number of cigarettes smoked per day by 20 (the equivalent of a pack) and multiplying this result by the number of years that the individual has smoked^12^.

Moreover, participants were asked about prior smoking. Thus, the smoking status was defined according to the categories of Reichert et al.^13^, with adaptation: current smoker (CS) for those that currently smoke; former smoker (FS) for those that have smoked in life and currently do not smoke; and non-smokers (NS) for those who have never smoked.

### Explanatory variables

The explanatory variables investigated were grouped into:

1. Sociodemographic and economic characteristics: gender, age, marital status, skin color, schooling, employment and economic class;
2. Behavioral characteristics and life habits: category of risk or exposure to HIV infection, alcohol use, illicit drugs, condom use and religious belief;
3. Clinical and laboratory characteristics: comorbidities, co-infections, signs and/or symptoms of anxiety or depression, clinical condition, CD4^+^ T lymphocyte count and viral load;
4. Features related to ART: therapeutic regimen, time of onset of ART, adherence, self-reported adverse drug reactions (ADR), number of self-reported ADR and HIV specialized service.

Alcohol use was measured using the following questions: “In your whole life, have you ever used alcohol?” (yes/no/did not want to inform/ ignored); and “How often do you drink alcohol?” (never/once a month or less/2 to 4 times a month/2 to 3 times a week/4 times a week/ 5 or more times a week/ ignored). No scales were used to classify alcohol consumption.

The use of crack, cocaine and marijuana substances was measured by the following questions: “In your whole life, have you ever used crack?”; “In your whole life, have you ever used cocaine?”; and “In your whole life, have you ever used marijuana?”, which could be answered with “yes,” “no,” “didn't want to report,” or “ignored.”

We used the Hospital Anxiety and Depression Scale (HADS), with validation in Brazil, to detect signs and/or symptoms of anxiety or depression^14^.

### Statistical Analysis

Epi Info® 3.5.4 software was used to type the questionnaire and the quality of data collection was verified by the Kappa method (k = 0.9549). Descriptive analysis was performed by frequency distribution for categorical variables, and measures of central tendency for quantitative variables, and Pearson's chi-squared test was then applied to analyze their association.

For the multivariate analysis, we performed a multinomial logistic regression with NS as reference category and all variables with p-value ≤ 0.20 from the univariate analysis. The stepwise method was used to obtain the final model, with significant variables (p ≤ 0.05) in at least one of the statuses (CS or FS). The magnitude of the association between the covariates and smoking was estimated by the odds ratio (OR), with a 95% confidence interval.

The goodness of fit of the multiple model was verified from the area under the ROC curve and from the data analysis by the SPSS® software version 22.

### Ethical aspects

The research was performed according to Resolution no. 466/2012 of the Brazilian National Health Council. The collected data were kept confidential, and the individuals’ identity was preserved. The study is part of the ECOART Project Effectiveness of ART in PLHIV, HIV/tuberculosis, HIV/leprosy or HIV/visceral leishmaniasis, followed in Belo Horizonte – approved by the Research Ethics Committee of the Universidade Federal Minas Gerais (CAAE 31192014.3.0000.5149) and by the participating services. All participants signed an informed consent form and an assent form in the case of children under 18 years of age.

## RESULTS

Of the 462 PLHIV interviewed, 27.7% were CS, 22.9% FS, and 49.4% NS. Table 1 shows the characteristics of the study population, stratified by smoking status.

**Table 1:** Characteristics of PLHIV assisted in Specialized Care Services in Belo Horizonte, according to smoking status, 2015-2017 (n = 462).

Characteristics		Total n = 462	Current smoker n = 128	Former smoker n = 106	Non-smoker n = 228	p^a^
		n (%)	n (%)	n (%)	n (%)	
Sociodemographic and economic						
	Gender (male)	376 (81.4)	97 (75.8)	88 (83.0)	191 (83.8)	0.157
Age (years)						0.083
	16–19	16 (3.5)	7 (5.5)	2 (1.9)	7 (3.1)	
	20–34	248 (53.7)	71 (55.5)	57 (53.8)	120 (52.6)	
	35–49	146 (31.6)	36 (28.1)	28 (26.4)	82 (36.0)	
	≥ 50	52 (11.3)	14 (10.9)	19 (17.9)	19 (8.3)	
Marital status (Single/divorced/widowed)		368 (79.7)	103 (80.5)	83 (78.3)	182 (79.8)	0.916
Race/skin color						**0.037**
	White	111 (24.3)	21 (16.8)	33 (31.1)	57 (25.2)	
	Non-white	346 (75.7)	104 (83.2)	73 (68.9)	169 (74.8)	
Schooling level (years)						**0.002**
	≤ 9	120 (26.0)	39 (30.5)	39 (37.1)	42 (18.4)	
	10–12	178 (38.6%)	51 (39.8)	37 (35.2)	90 (39.5)	
	13+	163 (35.4)	38 (29.7)	29 (27.6)	96 (42.1)	
Employment (yes)		270 (58.4)	61 (47.7)	54 (50.9)	155 (68.0)	**< 0.001**
Economic class						0.882
	C,D,E	289 (64.2)	80 (65.0)	67 (65.7)	142 (63.1)	
	A, B	161 (35.8)	43 (35.0)	35 (34.3)	83 (36.9)	
Behaviors and life habits						
HIV risk						0.160
	Heterosexual women	66 (14.3)	20 (15.6)	17 (16.0)	29 (12.7)	
	Heterosexual men	82 (17.7)	18 (14.1)	26 (24.5)	38 (16.7)	
	MSM	222 (48.1)	59 (46.1)	44 (41.5)	119 (52.2)	
	UDI/other^b^	21 (4.3)	10 (7.8)	5 (4.7)	6 (2.6)	
	Missing data	71 (15.4)	21 (16.4)	14 (13.2)	36 (15.8)	
	Alcohol use prior month (yes)	294 (64.1)	95 (74.8)	59 (55.7)	140 (61.9)	**0.007**
	Alcohol use in life (yes)	369 (80.0)	112 (87.5)	91 (85.8)	166 (73.1)	**0.001**
	Marijuana use in life (yes)	200 (43.6)	87 (68.5)	58 (55.2)	55 (24.2)	**< 0.001**
	Cocaine use in life (yes)	138 (30.1)	66 (52.0)	39 (37.1)	33 (14.5)	**< 0.001**
	Crack use in life (yes)	38 (8.3)	23 (18.1)	12 (11.4)	3 (1.3)	**< 0.001**
	I use condom prior month (yes)	252 (68.1)	64 (64.0)	46 (57.5)	142 (74.7)	**0.012**
	Religious belief (yes)	366 (79.6)	91 (72.2)	86 (81.1)	189 (82.9)	0.053
Clinics and laboratories						
	Comorbidity (yes)^c^	97 (21.0)	32 (25.0)	24 (22.6)	41 (18.0)	0.265
	Co-infection (yes)^d^	40 (8.7)	15 (11.7)	9 (8.7)	16 (7.0)	0.326
	Signs/symptoms of anxiety or depression (yes)	174 (37.7)	66 (51.6)	39 (36.8)	69 (30.3)	**< 0.001**
Clinical condition^e^						0.288
	Asymptomatic (category A)	291 (64.8)	84 (67.7)	64 (63.4)	143 (63.8)	
	Symptomatic (category B)	65 (14.5)	17 (13.7)	10 (9.9)	38 (17.0)	
	Aids (category C)	93 (20.7)	23 (18.5)	27 (26.7)	43 (19.2)	
CD4+ LT Count						0.535
	< 200 cells/ml	121 (26.2)	29 (22.7)	30 (28.3)	62 (27.2)	
	< 200 cells/mm	163 (35.3)	44 (34.4)	37 (34.9)	82 (36.0)	
	< 500 cells/mm	133 (28.8)	44 (34.4)	25 (23.6)	64 (28.1)	
	Missing data	45 (9.7)	11 (8.6)	14 (13.2)	20 (8.8)	
Viral load (copies/ml)						0.297
	≤ 100,000	302 (65.4)	92 (71.9)	62 (58.5)	148 (64.9)	
	> 100,000	115 (24.9)	25 (19.5)	31 (29.2)	59 (25.9)	
	Missing data	45 (9.7)	11 (8.6)	13 (12.3)	21 (9.2)	
ART-Related						
Therapeutic regimen						0.730
	TDF/3TC/EFV	291 (63.0)	75 (58.6)	68 (64.2)	148 (64.9)	
	TDF/3TC/DTG	143 (31.0)	43 (33.6)	33 (31.1)	67 (29.4)	
	Other schemes	28 (6.1)	10 (7.8)	5 (4.7)	13 (5.7)	
ART start time						0.921
	< 60 days	233 (50.4)	63 (49.2)	55 (51.9)	115 (50.4)	
	Adherence (yes)	197 (45.7)	47 (39.8)	45 (46.9)	105 (48.4)	0.313
	Self-reported ADR (yes)	370 (84.7)	104 (87.4)	84 (84.8)	182 (83.1)	0.578
	Self-reported ADR number (> 3)	203 (46.5)	60 (50.4)	52 (52.5)	91 (41.6)	0.114
Follow-up service						0.793
	I	174 (37.7)	48 (37.5)	43 (40.6)	83 (36.4)	
	II	176 (38.1)	45 (35.2)	39 (36.8)	92 (40.4)	
	III	112 (24.2)	35 (27.3)	24 (22.6)	53 (23.2)	

a Pearson's Chi-square test. Values with statistical significance are presented in bold.

LT-CD4^+^: T-CD4+ lymphocytes; MSM: men who have sex with men; IDI: injectable drug user; TDF: tenofovir; 3TC: lamivudine; EFV: efavirenz; DTG: dolutegravir; ART: antiretroviral therapy; ADR: adverse drug reaction; HIV: human immunodeficiency virus.

b other risks: hemophiliacs, transfusion and occupational.

c Comorbidities were considered those recorded at the first visit (*diabetes mellitus* – DM, systemic arterial hypertension - SAH, dyslipidemia, cancers or other).

d The co-infection considered in the first consultation corresponds to: tuberculosis, leishmaniasis, leprosy or other.

e Clinical classification according to the criteria of the Centers for Disease Control and Prevention adapted; A: asymptomatic, B: symptomatic, C: aids-defining symptoms.

Most participants were male (81.4%), young (up to 34 years old; 57.2%), employed at the time of the interview (58.4%) and had up to 9 years of schooling (26%). There was a high proportion of use of licit and illicit substances, mainly alcohol (80%) and marijuana (43.6%), and high prevalence of signs and/or symptoms of anxiety or depression (37.7%) in the population studied.

Comparing the three groups – CS, FS and NS – the results showed that most CS individuals were non-white, and only 47.7% of them were employed. The level of employability was higher among NS (68.0%). Regarding education, 30.5% of the CS had up to 9 years of schooling, 39.8% from 10 to 12 years, and 29.7% over 13 years. The highest level of education was among NS, among which 42.1% had 13 years or more of study. Regarding behavior and life habits, CS contributed to the highest percentage of reports of alcohol use in the previous month (74.8%) and life (87.5%), in addition to the use of illicit drugs (marijuana, cocaine and crack). Condom use in the previous month was higher among NS (74.7%), and only 64.0% of CS used it. Regarding clinical characteristics, CS were the ones that most reported signs and/or symptoms of anxiety or depression (51.6%). There were statistically significant differences among the groups (p-value ≤ 0.05) (Table 1).

Most PLHIV smokers were men (75.8%), non-white (83.2%), were unemployed (52.3%), had signs and/or symptoms of anxiety or depression (51.6%), used condoms in the previous month (64%) and have consumed alcoholic beverages (87.5%), marijuana (68.5%) and cocaine (52.0%). We observed that 30.5% of the PLHIV smokers had up to 9 years of schooling and 18.1% had already used crack (Table 1).

Although smoking started at approximately 17 years among CS and FS, the duration of cigarette use was longer among CS: 63.3% smoked for more than 10 years. Nevertheless, most of them were characterized by light (65.1%) and moderate (30.2%) consumption. The mast-year index was 10.05, on average (SD = 11.94) (Table 2).

**Table 2:** Tobacco use profile of current and former smoking PLHIV (n = 234).

Characteristics		Current smoker (n = 128)	Former smoker (n = 106)
		n (%)	n (%)
Smoking time (years)			
	≤ 10	47 (36.7)	68 (64.2)
	> 10	81 (63.3)	38 (35.8)
Cigarettes consumed per day			
	1–10 (light)	82 (65.1)	−
	11–20 (moderate)	38 (30.2)	−
	> 20(more than a pack) (heavy)	6 (4.8)	−
Pack-year index (SD)		26 (124)	−
Smoking onset age (years) – mean (SD)		17.2 (5.1)	17.4 (4.9)

To evaluate the factors associated with smoking, the characteristics of CS and FS were compared with the characteristics of NS in univariate and multivariate analyses (Tables 3 and 4).

**Table 3:** Univariate analysis of factors associated with current and former smoker in PLHIV attended in Specialized Care Services in Belo Horizonte/MG, 2015 – 2017, compared to nonsmokers) (n = 234).

Characteristics		Current smoker		p^a^	Former smoker		p^a^
		n = 128	%		n = 106	%	
Sociodemographic and economic							
Gender				**0.177**			0.863
	Male	97	25. 8		88	23.4	
	Female	31	36.0		18	20.9	
Age (years)				**0.116**			0.991
	≤ 33	74	31.1		52	21.8	
	> 33	54	24.1		54	24.1	
Marital status				0.884			0.749
	Single/divorced/widowed	103	28.0		83	22.6	
	Married/Stable Relationship	25	26.6		23	24.5	
Race/skin color				**0.071**			0.259
	White	21	18.9		33	29.7	
	Non-white	104	30.1		73	21.1	
Schooling level (years)							
	≤ 9	39	32.5	**0.004**	39	32.5	**< 0.001**
	10–12	51	28.7	0.167	37	20.8	0.285
	≥ 13	38	23.3		29	17.8	
Employment				**< 0.001**			**0.003**
	Yes	61	22.6		54	20.0	
	No	67	34.9		52	27.1	
Economic class							
	C, D, E	80	27.7	0.720	67	23.2	0.653
	A,B	43	26.7		35	21.7	
Behaviors and life habits							
HIV risk				0.249			**0.025**
	MSM	59	26.6		44	19.8	
	Other^b^	48	28.4		48	28.4	
Alcohol use prior month				**0.015**			0.276
	Yes	95	32.3		59	20.1	
	No	32	19.4		47	28.5	
Alcohol use in life				**0.002**			**0.011**
	Yes	112	30.4		91	24.7	
	No	16	17.4		15	16.3	
Marijuana use in life				**< 0.001**			**< 0.001**
	Yes	87	43.5		58	29.0	
	No	40	15.4		47	18.1	
Cocaine use in life				**< 0.001**			**< 0.001**
	Yes	66	47.8		39	28.3	
	No	61	19.0		66	20.6	
Crack use in life				**< 0.001**			**0.001**
	Yes	23	60.5		12	31.6	
	No	104	24.8		93	22.1	
I use condom prior month				**0.056**			**0.005**
	Yes	64	25.4		46	18.3	
	No	36	30.5		34	28.8	
Religious belief				**0.019**			0.694
	Yes	91	24.9		86	23.5	
	No	35	37.2		20	21.3	
Clinics and laboratories							
Comorbidity^c^				**0.117**			0.318
	Yes	32	33.0		24	24.7	
	No	96	26.3		82	22.5	
Co-infection^d^				**0.138**			0.608
	Yes	15	37.5		9	22.5	
	No	113	27.0		95	22.7	
Signs/symptoms of anxiety or depression				**< 0.001**			0.236
	Yes	66	37.9		39	22.4	
	No	62	21.5		67	23.3	
Clinical classification^e^				0.883			**0.128**
	No AIDS (A/B)	101	28.4		74	20.8	
	With aids (C)	23	24.7		27	29.0	
CD4+LT Count				0.334			0.628
	≤ 200 (cells/ml)	29	24.0		30	24.8	
	> 200 (cells/ml)	88	29.7		62	20.9	
Viral load (copies/ml)				**0.161**			0.399
	≤ 100,000	92	30.5		62	20.5	
	> 100,000	25	21.7		31	27.0	
ART-Related							
Therapeutic regimen							
	TDF/3TC/EFV	75	25.8		68	23.4	
	TDF/3TC/DTG	43	30.1	0.328	33	23.1	0.788
	Other schemes	10	35.7	0.347	5	17.9	0.745
ART start time				0.825			0.805
	≤ 60	63	27.0		55	23.6	
	> 60	65	28.4		51	22.3	
Adherence				**0.134**			0.805
	Yes	47	23.9		45	22.8	
	No	71	30.3		51	21.8	
Self-reported ADR				0.298			0.697
	Yes	104	28.1		84	22.7	
	No	15	22.4		15	22.4	
Self-reported ADR number				**0.118**			**0.069**
	≤ 3	59	25.2		47	20.1	
	>3	60	29.6		52	25.6	
Follow-up service							
	HEM	48	27.6	0.640	43	24.7	0.664
	CTA	45	25.6	0.290	39	22.2	0.832
	CTR	35	31.3		24	21.4	

a Pearson's Chi-square test. Values with statistical significance are presented in bold.

LT-CD4^+^: T-CD4+ lymphocytes; MSM: men who have sex with men; IDI: injectable drug user; TDF: tenofovir; 3TC: lamivudine; EFV: efavirenz; DTG: dolutegravir; ART: antiretroviral therapy; ADR: adverse drug reaction; HIV: human immunodeficiency virus.

b other risks: hemophiliacs, transfusion and occupational.

c Comorbidities were considered those recorded at the first visit (diabetes mellitus – DM, systemic arterial hypertension – SAH, dyslipidemia, cancers or other).

d The co-infection considered in the first consultation corresponds to: tuberculosis, leishmaniasis, leprosy or other.

e Clinical classification according to the criteria of the Centers for Disease Control and Prevention adapted; A: asymptomatic, B: symptomatic, C: aids-defining symptoms.

**Table 4:** Multivariate analysis of factors associated with smoking among current and former smokers compared to nonsmokers.

Characteristics	Current smoker^a^			Former smoker^b^		
	*Odds Ratio*	95%CI	p	*Odds Ratio*	95%CI	p
Gender (female)	2.18	(1.06 – 4.50)	**0.034**	0.85	(0.40–1.77)	0.655
Schooling (≤ 9 years *versus* ≥ 13 years)	2.35	(1.09–5.08)	**0.030**	4.72	(2.29–9.71)	**< 0.001**
Schooling (10–12 years *versus* ≥ 13 years)	1.80	(0.96–3.35)	0.065	1.73	(0.93–3.24)	0.084
Alcohol use in life (yes)	2.75	(1.31–5.77)	**0.008**	2.47	(1.22–4.98)	**0.011**
Marijuana use in life (yes)	5.52	(2.96–10.30)	**< 0.001**	3.66	(1.99–6.73)	**< 0.001**
Cocaine use in life (yes)	2.26	(1.18–4.34)	**0.014**	1.46	(0.74–2.86)	0.275
Crack use in life (yes)	8.61	(2.18–34.07)	**0.002**	4.84	(1.16–20.28)	**0.031**
Religious belief (yes)	0.50	(0.27–0.93)	**0.030**	0.82	(0.43–1.57)	0.548
Signs/symptoms of anxiety or depression (yes)	2.15	(1.26–3.68)	**0.005**	1.15	(0.66–1.98)	0.623

Values with statistical significance are presented in bold.

Area under the ROC curve = 0.788

Area under the ROC curve = 0.735

Table 3 shows that, when compared with NS, the variables with significance level equal to or less than 0.05 for CS were: having up to 9 years of schooling; not having a job; having used alcohol, marijuana, cocaine and crack; having no religious belief; signs and/or symptoms of anxiety or depression.

Regarding the FS, when compared with NS, the variables with significance level equal to or less than 0.05 were: having up to 9 years of schooling; not having a job; be in the CDE economy class; be at risk of HIV; use of alcohol, marijuana, cocaine and crack in life; and not having used condoms in the prior month (Table 3).

In the multivariate analysis, the following characteristics were independently associated with smoking: gender (female); schooling (≤ 9 years); have used alcohol, marijuana, cocaine and crack; signs and/or symptoms of anxiety or depression; and religious belief. Higher chances of being CS were found for females (OR = 2.18), people that had up to 9 years of schooling (OR = 2.35), and people that have used alcohol (OR = 2.75) and illicit drugs, and a strong association was observed with crack use (OR = 8.61), marijuana (OR = 5.52) and cocaine (OR = 2.26). An even greater chance of being a smoker was observed in people with signs and/or symptoms of anxiety or depression (OR = 2.15), and less chance of being a smoker in people with religious belief (OR = 0.50) (Table 4).

In the comparison between FS and NS, the characteristics associated with smoking were: having up to 9 years of schooling (OR = 4.72) and having already used alcohol (OR = 2.47), marijuana (OR = 3.66) and crack (OR = 4.84) (Table 4).

## DISCUSSION

Our study showed a high prevalence of smoking among PLHIV. T obacco consumption among CS is characterized by prolonged use (over 10 years) and, mostly, light to moderate use. Current smoking was strongly associated with gender, schooling, use of licit and illicit substances, and presence of signs and/or symptoms of anxiety or depression, whereas FS was associated only with schooling and substance use. Moreover, having a religious belief was a protective factor against current smoking.

The characteristics of CS in our study are in accordance with epidemiological profiles and national and international data^3^^–^^8^^,^^15^^,^^16^. This population is mainly composed of men, young people, people with a lower level of education, with high consumption of alcohol and illicit drugs, and who presented signs and/or symptoms of anxiety or depression. Smoking and time of cigarette use in our study were also similar to that observed in other metropolises in Brazil^5^^,^^6^ and in international literature, i.e., most CS smoked up to 10 cigarettes (half pack) per day and for more than 10 years^4^.

The prevalence of smoking was 27.7%, similar to that reported in a few cohort studies conducted in the country: 28.9% among 1,815 PLHIV in Recife^5^; 29.9% among 2,775 PLHIV in Rio de Janeiro^6^, and 33.6% among 440 PLHIV in a reference center in Belo Horizonte^7^. In contrast, we emphasize that the prevalence of smoking is 40% to 67% in international cohorts of HIV-PLHIV^2^^,^^15^^,^^17^.

The lower prevalence of smoking in Brazil may be related to the effects of the National Tobacco Control Policy (PNCT)), instituted in the country since the late 1980s, which led to a general decrease in smoking in the Brazilian population^8^. Nevertheless, the prevalence of current smoking among PLHIV found in our study is much higher than that of the general Brazilian population, estimated at 10.1%^8^. This result shows the need for prioritizing smoking cessation interventions directed to PLHIV.

PLHIV users of alcohol and illicit drugs were more likely to be smokers and former smokers compared to NS, and crack use was strongly associated with smoking. The association between alcohol, illicit drugs and smoking in PLHIV has been widely reported in the literature, and these characteristics are associated with worse clinical outcomes among PLHIV^3^^–^^7^^,^^18^^,^^19^.

In another study of the ECOART Project that followed PLHIV within 12 months of ART, the results obtained from medical records data showed that smoking and illicit drug use reduced the chance of achieving viral suppression^7^. As discussed in the literature, the cytochrome P450 pathway is common to the metabolism of nicotine, alcohol, illicit drugs and medications. This pathway plays an important role in drug interactions in PLHIV that use those substances due to the ability of enzymatic induction and inhibition. It is emphasized that the high prevalence of dependence and abuse of licit and illicit substances in PLHIV is associated with non-aptitude to ART, and consequently, to the failure of viral suppression^20^.

In Vietnam, a survey of 409 PLHIV smokers showed that around 37% and 69% were alcohol and drug users, respectively, and 41% had anxiety^21^.

In our study, PLHIV with signs and/or symptoms of anxiety or depression were twice as likely to be smokers. These findings are in agreement with the cohort of Rio de Janeiro, in which depression was independently associated with CS when compared with NS (OR = 1.38)^6^.

As in our study, other studies reported high rates of mental suffering among PLHIV (between 25.9% and 66.3%), with depression and anxiety being the most common problems^7^^,^^22^. The presence of these comorbidities was also observed in a cross-sectional study conducted in China with 360 PLHIV, comparing men and women, in which the overall prevalence of depressive and anxious symptoms was 66.3% and 45.6%, respectively^22^.

Another study showed that frequent depressive disorder and emotional distress influence the high rates of smoking in PLHIV^23^. There was an association of greater abstinence from the measures of general distress, based on anxiety and depression of aversive affective states (anger, anxiety, depression, confusion and fatigue), as well as generalized anxiety disorder, a syndrome defined by excessive concern, tension, fatigue, concentration problems and irritability^23^.

The difficulty of stopping smoking reported by smokers is that, in addition to having to endure withdrawal caused by physical dependence, the other difficulties that have been covered by this habit emerge with all force, because smokers see smoking as a complement of themselves, which provides relief, pleasure and relaxation^a^. Cessation of tobacco use in PLHIV tends to be more difficult, since different components of anxiety and depression are associated with withdrawal from this substance^23^.

Moreover, having a religious belief was a protective factor against current smoking. A study with data from the German Socioeconomic Panel (1998–2006) to estimate the relationship between religion and smoking concluded that religiosity significantly affects smoking behavior, suggesting that atheists are 13% to 19% more likely to smoke than religious individuals. According to the authors, religion functions as a channel, in which it is possible for society to impose rules, and it is relevant for public health policy makers to consider religiosity when directing anti-smoking interventions^24^.

Smoking cessation programs are recommended to engage members of the smoker's social network due to their influence on smoking-related behavior, with a view to the greater effectiveness of these programs^4^.

Regarding the limitations of our study, we can cite the lack of biochemical verification, since we only considered only the participants report of cigarette use. The social desirability bias may have been present, possibly resulting in underestimated prevalence of smoking, because it is understood as an inappropriate behavior. Moreover, the data obtained from the Siclom and Siscel information systems could be incomplete. As a strong point, we cite the high quality of data collection and the robustness of the final model.

Smoking and its cessation in PLHIV population must be further studies to enable a faster transition from finding risks to effective interventions.

## CONCLUSIONS

In our study, we observed that a higher chance of being CS among PLHIV was found for individuals that have used crack, marijuana, alcohol and/or cocaine, with lower schooling and with signs and/or symptoms of anxiety or depression. For people with religious beliefs, the chance of being a smoker was lower.

Our study corroborates previous research that indicated that smoking is highly prevalent among PLHIV. The prevalence of smoking found was almost three times compared to the general Brazilian population, showing the need for HIV specialized services to prioritize smoking cessation interventions. These interventions should consider alcohol and illicit drug abuse and be targeted especially to young people, with low schooling and with signs and/or symptoms of anxiety or depression.

## Footnotes

a Charran IM. Interview: Ivone Maria Charran. Interview given to Ebah. S.l.: Ebah; ago 2018. [cited Aug 31, 2018]. Available from: http://www.ebah.com.br/content/ABAAABr0sAD/smoking
