# Supplementary material for: Prevalence of smoking and associated factors in people living with HIV undergoing treatment
Source: Rev Saude Publica. 2020 Nov 4;54:108. doi: 10.11606/s1518-8787.2020054001828 (PMC7647468; doi:10.11606/s1518-8787.2020054001828)
Supplement: Translation

# Translation: Prevalência e fatores associados ao tabagismo em pessoas vivendo com HIV em tratamento

**Authors:** Luciane de Souza Leal Teixeira, Maria das Graças Braga Ceccato, Wânia da Silva Carvalho, Juliana de Oliveira Costa, Palmira de Fátima Bonolo, Jullye Campos Mendes, Micheline Rosa Silveira

**Affiliations:** I Belo HorizonteMG Brasil Universidade Federal de Minas Gerais. Faculdade de Farmácia. Programa de Pós-Graduação em Medicamentos e Assistência Farmacêutica. Belo Horizonte, MG, Brasil; II Belo HorizonteMG Brasil Universidade Federal de Minas Gerais. Faculdade de Farmácia. Departamento de Farmácia Social. Belo Horizonte, MG, Brasil; III Sydney Australia Centre for Big Data Research in Health. Faculty of Medicine. UNSW Sydney, Sydney, Australia; IV Belo HorizonteMG Brasil Universidade Federal de Minas Gerais. Faculdade de Medicina. Departamento de Medicina Preventiva e Social. Belo Horizonte, MG, Brasil; V Universidade Federal de Minas Gerais. Faculdade de Medicina. Programa de Pós-Graduação em Saúde Pública. Belo Horizonte, MG, Brasil

**Keywords:** Sobreviventes de Longo Prazo ao HIV, Fatores de Risco, Tabagismo, Alcoolismo, Transtornos Relacionados ao Uso de Substâncias, Estudos Transversais

## Abstract

**OBJETIVO:**

Estimar a prevalência do tabagismo e avaliar os fatores a ele associados em pessoas vivendo com HIV (PVHIV).

**MÉTODOS:**

Trata-se de estudo transversal de uma coorte prospectiva concorrente com 462 indivíduos em início de terapia antirretroviral atendidos em três serviços de assistência especializada ao HIV/aids em Belo Horizonte entre 2015 e 2017. Os *status* de tabagismo utilizados foram: fumante atual (FA), ex-fumante (EF) e não fumante (NF). Realizou-se regressão logística multinomial, sendo NF a categoria de referência.

**RESULTADOS:**

A maioria dos participantes eram homens (81,4%), jovens (de até 34 anos; 57,2%) e não brancos (75,7%). Do total de indivíduos, 27,7% eram FA, 22,9% EF, e 49,4% NF. A maioria dos tabagistas eram fumantes leves (65,1%), consumiam até 10 cigarros por dia e fumavam havia mais de 10 anos (63,3%), tendo começado em média aos 17,2 anos de idade (DP = 5,1). Na análise multivariada, maiores chances de ser FA se associaram a: ser do sexo feminino, ter até 9 anos de escolaridade, usar ou já ter usado álcool e drogas ilícitas (maconha, cocaína e crack) e apresentar sinais e/ou sintomas de ansiedade ou depressão. Maiores chances de ser EF se associaram a ter até 9 anos de escolaridade e usar ou já ter usado álcool e drogas e ilícitas (maconha e crack).

**CONCLUSÕES:**

Os resultados mostram que o tabagismo é altamente prevalente entre PVHIV, indicando a necessidade de os serviços de assistência especializada em HIV priorizarem intervenções a fim de cessá-lo, com abordagem sobre o uso de álcool e drogas ilícitas, especialmente voltadas para pessoas jovens, com baixa escolaridade e com sinais e/ou sintomas de ansiedade ou depressão.

## INTRODUÇÃO

O tabagismo é a principal causa de morte evitável no mundo e, em pessoas vivendo com HIV (PVHIV), está associado a significativa morbimortalidade^1^. Estima-se que a prevalência mundial do tabagismo em PVHIV seja de 40% a 70% e, na população geral, em torno de 20%^2^^–^^4^.

No Brasil, estudos prévios estimaram que a prevalência de tabagismo em PVHIV variou de 28,9% a 33,6%^5^^–^^7^, enquanto na população geral acima de 18 anos esse índice é de 10,1%^8^ – ou seja, é cerca de três vezes maior em PVHIV. Apesar disso, poucos estudos em países em desenvolvimento têm analisado os fatores associados ao tabagismo em PVHIV.

O tabagismo é uma condição de saúde epidêmica que causa dependência física, psicológica e comportamental, atribuída especialmente à nicotina^9^. Os componentes derivados da queima do cigarro causam alterações morfofuncionais de macrófagos, linfócitos B e T (CD4 e CD8) e linfocinas, além de promover a expressão do gene HIV-1 e, consequentemente, a aids^6^^,^^10^^,^^11^. A menor resposta ao uso de terapia antirretroviral (TARV), com consequente supressão viral prejudicada em PVHIV fumantes, envolve o mecanismo da variante CYP1A1-M1 do citocromo P450 (CYP)^10^.

Tanto os componentes do cigarro quanto demais fatores que influenciam o comportamento das PVHIV podem estar associados ao insucesso da TARV nessa população fumante. Esses fatores incluem baixa adesão ao tratamento, uso de substâncias lícitas e ilícitas, depressão e ansiedade, e alterações da função imunológica^10^^,^^7^.

Os esquemas de TARV disponibilizados pelo Sistema Único de Saúde no Brasil foram alterados recentemente, seguindo recomendações internacionais^9^. Diante desse cenário, torna-se importante compreender os fatores associados e a prevalência de tabagismo em PVHIV em tratamento para planejar intervenções efetivamente direcionadas a esses indivíduos.

O objetivo deste estudo foi estimar a prevalência e os fatores associados ao tabagismo, bem como o perfil de uso de tabaco de PVHIV iniciando a TARV em serviços públicos de referência em Belo Horizonte (MG).

## MÉTODOS

Fez-se um estudo transversal de dados de entrevistas basais da coorte do Projeto ECOART, realizado em Belo Horizonte (MG), Brasil. A seleção da amostra foi não aleatória, sendo composta por 462 indivíduos em início de TARV identificados no Sistema de Controle Logístico de Medicamentos (Siclom) e no Sistema de Controle de Exames Laboratoriais da Rede Nacional de Contagem de Linfócitos CD4^+^/CD8^+^ e Carga Viral do HIV (Siscel), os quais responderam a um questionário estruturado aplicado face a face por equipe treinada (Figura 1).

**Figura 1:** Diagrama de elegibilidade das pessoas incluídas no estudo. Belo Horizonte, MG, 2017.

Os indivíduos foram recrutados de setembro de 2015 a outubro de 2017 em três serviços públicos de assistência especializada em HIV/aids, responsáveis por dispensar TARV para aproximadamente 80% das PVHIV no município de Belo Horizonte. Foram incluídas pessoas com idade igual ou superior a 13 anos. O Serviço I é um serviço de assistência especializada (SAE) em HIV, que oferece cuidados intensivos hospitalares e ambulatoriais. O Serviço II é um centro de testagem e aconselhamento (CTA), e o Serviço III é um SAE – ambos vinculados à Prefeitura de Belo Horizonte –, que oferecem assistência médica interdisciplinar.

A variável-resposta foi fumar cigarro no momento da entrevista face-a-face, sendo mensurada pela pergunta: “Atualmente você fuma cigarro?”, cuja resposta poderia ser “sim”, “não” ou “ignorado”.

Os fumantes foram classificados pelo número de cigarros fumados ao dia em três níveis: uso leve (até 10), moderado (de 10 a 20) ou pesado (acima de 20), de acordo com o autorrelato. O número de anos-maço foi definido dividindo-se o número de cigarros fumados ao dia por 20 (o equivalente a um maço) e multiplicando-se este resultado pelo número de anos que o indivíduo fumou^12^.

Adicionalmente, os participantes foram questionados sobre já ter fumado no passado. Desta forma, o *status* de tabagismo foi definido segundo as categorias de Reichert et al.^13^, com adaptação: fumante atual (FA) para quem fuma atualmente; ex-fumante (EF) para quem já fumou na vida e atualmente não fuma; e não fumante (NF) para quem nunca fumou.

### Variáveis Explicativas

As variáveis explicativas investigadas foram agrupadas em:

1. Características sociodemográficas e econômicas: sexo, idade, estado civil, cor da pele, escolaridade, emprego e classe econômica;
2. Características comportamentais e hábitos de vida: risco de HIV, uso de álcool, drogas ilícitas, preservativo e crença religiosa;
3. Características clínicas e laboratoriais: comorbidades, coinfecções, sinais e/ou sintomas de ansiedade ou depressão, condição clínica, contagem de linfócitos T CD4^+^ e carga viral;
4. Características relacionadas à TARV: esquema terapêutico, tempo de início da TARV, adesão, reações adversas a medicamentos (RAM) autorrelatadas, número de RAM autorrelatadas e serviço de acompanhamento.

O uso de álcool foi mensurado por meio das perguntas: “Em toda a sua vida, alguma vez você usou de álcool?”, cuja resposta poderia ser “sim”, “não”, “não quis informar” ou “ignorado”; e “Com que frequência você costuma consumir bebida alcoólica?” (“nunca”, “uma vez por mês ou menos”, “2 a 4 vezes por mês”, “2 a 3 vezes por semana”, “4 vezes por semana”, “5 ou mais vezes por semana”, ou “ignorado”). Não foram utilizadas neste trabalho escalas para classificação de consumo de álcool.

O uso das substâncias crack, cocaína e maconha foi mensurado pelas perguntas: “Em toda a sua vida, alguma vez você fez uso de crack?”; “Em toda a sua vida, alguma vez você fez uso de cocaína?”; e “Em toda a sua vida, alguma vez você fez uso de maconha?”, que poderiam ser respondidas com “sim”, “não”, “não quis informar” ou “ignorado”.

A Escala Hospitalar de Ansiedade e Depressão (HADS), com validação no Brasil, foi utilizada para detectar sinais e/ou sintomas de ansiedade ou depressão^14^.

### Análise Estatística

Foi utilizado o software Epi Info® 3.5.4 para digitação das respostas, cuja qualidade foi verificada pelo método Kappa (k = 0,9549). A análise descritiva foi realizada por distribuição de frequências para variáveis categóricas, e medidas de tendência central para as variáveis quantitativas, sendo aplicado em seguida o teste qui-quadrado de Pearson para análise da associação entre ambas.

Para a análise multivariada, realizou-se regressão logística multinomial, a categoria de referência foi NF, e entraram no modelo as variáveis com valor-*p* ≤ 0,20 na análise univariada. Utilizou-se o método *stepwise* para obtenção do modelo final, com variáveis significativas (p ≤ 0,05) em pelo menos um dos *status* (FA ou EF). A magnitude da associação entre as covariáveis e o tabagismo foi estimada pela *odds ratio* (OR), com intervalo de confiança de 95%.

A qualidade do ajuste do modelo múltiplo foi verificada a partir da área sob a curva ROC e da análise dos dados pelo software SPSS® versão 22.

### Aspectos Éticos

A pesquisa foi realizada de acordo com a Resolução nº 466/2012 do Conselho Nacional de Saúde. Os dados coletados foram mantidos em sigilo, e a identificação dos indivíduos foi preservada. O estudo integra o Projeto ECOART – “Efetividade da TARV em PVHIV, HIV/tuberculose, HIV/hanseníase ou HIV/leishmaniose visceral, acompanhados em Belo Horizonte” –, aprovado pelo Comitê de Ética em Pesquisa da Universidade Federal de Minas Gerais (CAAE 31192014.3.0000.5149) e pelos serviços participantes. Todos os participantes assinaram termo de consentimento livre e esclarecido e, no caso de menores de 18 anos, termo de assentimento.

## RESULTADOS

Das 462 PVHIV entrevistadas, 27,7% eram FA, 22,9% EF, e 49,4% NF. Na Tabela 1 são apresentadas as características da população do estudo, estratificada por *status* de tabagismo.

**Tabela 1:** Características das PVHIV atendidas em Serviços de Assistência Especializada em Belo Horizonte, segundo o *status* de tabagismo, 2015–2017. (n = 462)

Características		Total n = 462	Fumante atual n = 128	Ex-fumante n = 106	Não fumante n = 228	p^a^
		n (%)	n (%)	n (%)	n (%)	
Sociodemográficas e econômicas						
Sexo (masc.)		376 (81,4)	97 (75,8)	88 (83,0)	191 (83,8)	0,157
Idade (anos)						0,083
	16–19	16 (3,5)	7 (5,5)	2 (1,9)	7 (3,1)	
	20-34	248 (53,7)	71 (55,5)	57 (53,8)	120 (52,6)	
	35-49	146 (31,6)	36 (28,1)	28 (26,4)	82 (36,0)	
	≥ 50	52 (11,3)	14 (10,9)	19 (17,9)	19 (8,3)	
Estado civil (Solteiro/divorciado/viúvo)		368 (79,7)	103 (80,5)	83 (78,3)	182 (79,8)	0,916
Raça/cor da pele						**0,037**
	Branco	111 (24,3)	21 (16,8)	33 (31,1)	57 (25,2)	
	Não branco	346 (75,7)	104 (83,2)	73 (68,9)	169 (74,8)	
Escolaridade (anos)						**0,002**
	≤ 9	120 (26,0)	39 (30,5)	39 (37,1)	42 (18,4)	
	10–12	178 (38,6%)	51 (39,8)	37 (35,2)	90 (39,5)	
	13+	163 (35,4)	38 (29,7)	29 (27,6)	96 (42,1)	
Emprego (sim)		270 (58,4)	61 (47,7)	54 (50,9)	155 (68,0)	**< 0,001**
Classe econômica						0,882
	C, D, E	289 (64,2)	80 (65,0)	67 (65,7)	142 (63,1)	
	A, B	161 (35,8)	43 (35,0)	35 (34,3)	83 (36,9)	
Comportamentais e hábitos de vida						
Risco HIV						0,160
	Mulheres heterossexuais	66 (14,3)	20 (15,6)	17 (16,0)	29 (12,7)	
	Homens heterossexuais	82 (17,7)	18 (14,1)	26 (24,5)	38 (16,7)	
	HSH	222 (48,1)	59 (46,1)	44 (41,5)	119 (52,2)	
	UDI/outros^b^	21 (4,3)	10 (7,8)	5 (4,7)	6 (2,6)	
	Dados faltantes	71 (15,4)	21 (16,4)	14 (13,2)	36 (15,8)	
	Uso de álcool último mês (sim)	294 (64,1)	95 (74,8)	59 (55,7)	140 (61,9)	**0,007**
	Uso de álcool na vida (sim)	369 (80,0)	112 (87,5)	91 (85,8)	166 (73,1)	**0,001**
	Uso de maconha na vida (sim)	200 (43,6)	87 (68,5)	58 (55,2)	55 (24,2)	**< 0,001**
	Uso de cocaína na vida (sim)	138 (30,1)	66 (52,0)	39 (37,1)	33 (14,5)	**< 0,001**
	Uso de crack na vida (sim)	38 (8,3)	23 (18,1)	12 (11,4)	3 (1,3)	**< 0,001**
	Uso preservativo último mês (sim)	252 (68,1)	64 (64,0)	46 (57,5)	142 (74,7)	**0,012**
	Crença religiosa (sim)	366 (79,6)	91 (72,2)	86 (81,1)	189 (82,9)	0,053
Clínicas e laboratoriais						
	Comorbidade (sim)^c^	97 (21,0)	32 (25,0)	24 (22,6)	41 (18,0)	0,265
	Coinfecção (sim)^d^	40 (8,7)	15 (11,7)	9 (8,7)	16 (7,0)	0,326
	Sinais/sintomas de ansiedade ou depressão (sim)	174 (37,7)	66 (51,6)	39 (36,8)	69 (30,3)	**< 0,001**
Condição clínica^e^						0,288
	Assintomático (categoria A)	291 (64,8)	84 (67,7)	64 (63,4)	143 (63,8)	
	Sintomático (categoria B)	65 (14,5)	17 (13,7)	10 (9,9)	38 (17,0)	
	Aids (categoria C)	93 (20,7)	23 (18,5)	27 (26,7)	43 (19,2)	
	Contagem LT CD4+					0,535
	< 200 células/ml	121 (26,2)	29 (22,7)	30 (28,3)	62 (27,2)	
	200–500 células/ml	163 (35,3)	44 (34,4)	37 (34,9)	82 (36,0)	
	> 500 células/ml	133 (28,8)	44 (34,4)	25 (23,6)	64 (28,1)	
	Dados faltantes	45 (9,7)	11 (8,6)	14 (13,2)	20 (8,8)	
Carga viral (cópias/ml)						0,297
	≤ 100 mil	302 (65,4)	92 (71,9)	62 (58,5)	148 (64,9)	
	> 100 mil	115 (24,9)	25 (19,5)	31 (29,2)	59 (25,9)	
	Dados faltantes	45 (9,7)	11 (8,6)	13 (12,3)	21 (9,2)	
Relacionadas à TARV						
Esquema terapêutico						0,730
	TDF/3TC/EFV	291 (63,0)	75 (58,6)	68 (64,2)	148 (64,9)	
	TDF/3TC/DTG	143 (31,0)	43 (33,6)	33 (31,1)	67 (29,4)	
	Outros esquemas	28 (6,1)	10 (7,8)	5 (4,7)	13 (5,7)	
Tempo de início TARV						0,921
	< 60 dias	233 (50,4)	63 (49,2)	55 (51,9)	115 (50,4)	
	Adesão (sim)	197 (45,7)	47 (39,8)	45 (46,9)	105 (48,4)	0,313
	RAM autorrelatadas (sim)	370 (84,7)	104 (87,4)	84 (84,8)	182 (83,1)	0,578
	Número RAM autorrelatadas (> 3)	203 (46,5)	60 (50,4)	52 (52,5)	91 (41,6)	0,114
Serviço de acompanhamento						0,793
	I	174 (37,7)	48 (37,5)	43 (40,6)	83 (36,4)	
	II	176 (38,1)	45 (35,2)	39 (36,8)	92 (40,4)	
	III	112 (24,2)	35 (27,3)	24 (22,6)	53 (23,2)	

a Teste qui-quadrado de *Pearson.* Valores com significância estatística estão apresentados em negrito.

LT-CD4^+^: linfócitos T-CD4^+^; HSH: homens que fazem sexo com homens; UDI: usuário de droga injetável; TDF: tenofovir; 3TC: lamivudina; EFV: efavirenz; DTG: dolutegravir; TARV: terapia antirretroviral; RAM: reação adversa a medicamento; HIV: *human immunodeficiency virus*.

b Outros riscos: hemofílicos, transfusão e ocupacional.

c As comorbidades foram consideradas as registradas na primeira consulta (*diabetes mellitus* - DM, hipertensão arterial sistêmica - HAS, dislipidemia, cânceres ou outra).

d A coinfecção considerada na primeira consulta corresponde a: tuberculose, leishmaniose, hanseníase ou outra.

e Classificação clínica segundo os critérios do *Centers for Disease Control and Prevention* adaptados; A: assintomático, B: sintomático, C: sintomas definidores de aids.

A maioria dos participantes eram do sexo masculino (81,4%), jovens (de até 34 anos; 57,2%), estavam empregados no momento da entrevista (58,4%) e tinham até 9 anos de escolaridade (26%). Observou-se elevada proporção de uso de substâncias lícitas e ilícitas, principalmente álcool (80%) e maconha (43,6%), e elevada prevalência de sinais e/ou sintomas de ansiedade ou depressão (37,7%) na população estudada.

Comparando-se os três grupos – FA, EF e NF –, os resultados mostraram que a maioria dos indivíduos FA eram não brancos, e apenas 47,7% deles tinham emprego. O nível de empregabilidade foi maior entre os NF (68,0%). Quanto à escolaridade, 30,5% dos FA tinham até 9 anos de escolaridade, 39,8% de 10 a 12 anos, e 29,7% acima de 13 anos. O maior nível de escolaridade foi entre os NF, entre os quais 42,1% acumulavam 13 anos ou mais de estudo. Em relação a comportamento e hábitos de vida, os FA contribuíram com o maior percentual de relatos de uso de álcool no último mês (74,8%) e na vida (87,5%), além do uso de drogas ilícitas (maconha, cocaína e crack). O uso de preservativo no último mês foi maior entre os NF (74,7%), e apenas 64,0% dos FA utilizaram-no. Quanto às características clínicas, os FA foram os que mais relataram sinais e/ou sintomas de ansiedade ou depressão (51,6%). Houve diferenças estatisticamente significativas entre os grupos (valor-*p* ≤ 0,05) (Tabela 1).

A maioria das PVHIV fumantes eram homens (75,8%), não brancas (83,2%), estavam desempregadas (52,3%), apresentavam sinais e/ou sintomas de ansiedade ou depressão (51,6%), usaram preservativo no último mês (64%) e, ao longo da vida, consumiram bebida alcoólica (87,5%), maconha (68,5%) e cocaína (52,0%). Foi observado que 30,5% das PVHIV fumantes tinham até 9 anos de escolaridade e 18,1% já usaram crack (Tabela 1).

Embora o início do tabagismo tenha ocorrido aproximadamente aos 17 anos entre FA e EF, a duração do uso de cigarro foi maior entre os FA: 63,3% fumavam por um período maior que 10 anos. Apesar disso, a maior parte foi caracterizada por um consumo leve (65,1%) e moderado (30,2%). O índice de maços-ano foi de 10,05, em média (DP = 11,94) (Tabela 2).

**Tabela 2:** Perfil de uso do tabaco das PVHIV fumante atual e ex-fumante (n = 234).

Características		Fumante atual (n = 128)	Ex-fumante (n = 106)
		n (%)	n (%)
Tempo de fumo (anos)			
	≤ 10	47 (36,7)	68 (64,2)
	> 10	81 (63,3)	38 (35,8)
Cigarros consumidos por dia			
	1–10 (leve)	82 (65,1)	−
	11–20 (moderado)	38 (30,2)	−
	> 20 (acima de um maço) (pesado)	6 (4,8)	−
Índice maços-ano (DP)		26 (124)	−
Idade de início tabagismo (anos) - média (DP)		17,2 (5,1)	17,4 (4,9)

Para avaliar os fatores associados ao tabagismo, foram comparadas as características dos FA e dos EF com as características dos NF, em análises univariadas e multivariadas (Tabelas 3 e 4).

**Tabela 3:** Análise univariada dos fatores associados ao fumante atual e ex-fumante em PVHIV atendidas em Serviços de Assistência Especializada em Belo Horizonte/MG, 2015–2017, comparados aos não fumantes (n = 234).

Características		Fumante atual		p^a^	Ex-fumante		p^a^
		n = 128	%		n = 106	%	
Sociodemográficas e econômicas							
Sexo				**0,177**			0,863
	Masculino	97	25, 8		88	23,4	
	Feminino	31	36,0		18	20,9	
Idade (anos)				**0,116**			0,991
	≤ 33	74	31,1		52	21,8	
	> 33	54	24,1		54	24,1	
Estado civil				0,884			0,749
	Solteiro/divorciado/viúvo	103	28,0		83	22,6	
	Casado-união	25	26,6		23	24,5	
Raça/cor da pele				**0,071**			0,259
	Branco	21	18,9		33	29,7	
	Não branco	104	30,1		73	21,1	
Escolaridade (anos)							
	≤ 9	39	32,5	**0,004**	39	32,5	**< 0,001**
	10 – 12	51	28,7	0,167	37	20,8	0,285
	≥ 13	38	23,3		29	17,8	
Emprego				**< 0,001**			**0,003**
	Sim	61	22,6		54	20,0	
	Não	67	34,9		52	27,1	
Classe econômica							
	C, D, E	80	27,7	0,720	67	23,2	0,653
	A,B	43	26,7		35	21,7	
Comportamentais e hábitos de vida							
Risco HIV				0,249			**0,025**
	HSH	59	26,6		44	19,8	
	Outros^b^	48	28,4		48	28,4	
Uso de álcool último mês				**0,015**			0,276
	Sim	95	32,3		59	20,1	
	Não	32	19,4		47	28,5	
Uso de álcool na vida				**0,002**			**0,011**
	Sim	112	30,4		91	24,7	
	Não	16	17,4		15	16,3	
Uso de maconha na vida				**< 0,001**			**< 0,001**
	Sim	87	43,5		58	29,0	
	Não	40	15,4		47	18,1	
Uso de cocaína na vida				**< 0,001**			**< 0,001**
	Sim	66	47,8		39	28,3	
	Não	61	19,0		66	20,6	
Uso de crack na vida				**< 0,001**			**0,001**
	Sim	23	60,5		12	31,6	
	Não	104	24,8		93	22,1	
Uso de preservativo último mês				**0,056**			**0,005**
	Sim	64	25,4		46	18,3	
	Não	36	30,5		34	28,8	
Crença religiosa				**0,019**			0,694
	Sim	91	24,9		86	23,5	
	Não	35	37,2		20	21,3	
Clínicas e laboratoriais							
Comorbidade^c^				**0,117**			0,318
	Sim	32	33,0		24	24,7	
	Não	96	26,3		82	22,5	
Coinfecção^d^				**0,138**			0,608
	Sim	15	37,5		9	22,5	
	Não	113	27,0		95	22,7	
Sinais/sintomas ansiedade ou depressão				**< 0,001**			0,236
	Sim	66	37,9		39	22,4	
	Não	62	21,5		67	23,3	
Classificação clínica^e^				0,883			**0,128**
	Sem aids (A/B)	101	28,4		74	20,8	
	Com aids (C)	23	24,7		27	29,0	
Contagem LT CD4+				0,334			0,628
	≤ 200 (células/ml)	29	24,0		30	24,8	
	>200 (células/ml)	88	29,7		62	20,9	
Carga Viral (cópias/ml)				**0,161**			0,399
	≤ 100 mil	92	30,5		62	20,5	
	> 100 mil	25	21,7		31	27,0	
Relacionadas à TARV							
Esquema terapêutico							
	TDF/3TC/EFV	75	25,8		68	23,4	
	TDF/3TC/DTG	43	30,1	0,328	33	23,1	0,788
	Outros esquemas	10	35,7	0,347	5	17,9	0,745
Tempo de início TARV (dias)				0,825			0,805
	≤ 60	63	27,0		55	23,6	
	> 60	65	28,4		51	22,3	
Adesão				**0,134**			0,805
	Sim	47	23,9		45	22,8	
	Não	71	30,3		51	21,8	
RAM autorrelatadas				0,298			0,697
	Sim	104	28,1		84	22,7	
	Não	15	22,4		15	22,4	
Número RAM autorrelatadas				**0,118**			**0,069**
	≤ 3	59	25,2		47	20,1	
	>3	60	29,6		52	25,6	
Serviço de acompanhamento							
	HEM	48	27,6	0,640	43	24,7	0,664
	CTA	45	25,6	0,290	39	22,2	0,832
	CTR	35	31,3		24	21,4	

a Teste qui-quadrado de *Pearson.* Valores com significância estatística estão apresentados em negrito.

LT-CD4^+^: linfócitos T-CD4^+^; HSH: homens que fazem sexo com homens; UDI: usuário de droga injetável; TDF: tenofovir; 3TC: lamivudina; EFV: efavirenz; DTG: dolutegravir; TARV: terapia antirretroviral; RAM: reação adversa a medicamento; HIV: *human immunodeficiency Virus*.

b Outros riscos: hemofílicos, transfusão e ocupacional.

c As comorbidades foram consideradas as registradas na primeira consulta (*diabetes mellitus* - DM, hipertensão arterial sistêmica - HAS, dislipidemia, cânceres ou outra).

d A coinfecção considerada na primeira consulta corresponde a: tuberculose, leishmaniose, hanseníase ou outra.

e Classificação clínica segundo os critérios do *Centers for Disease Control and Prevention* adaptados; A:assintomático, B: sintomático, C: sintomas definidores de aids.

**Tabela 4:** Análise multivariada dos fatores associados ao tabagismo entre fumantes atuais e ex-fumantes em comparação aos não fumantes.

Características	Fumante atual^a^			Ex-fumante^b^		
	*Odds ratio*	IC95%	p	*Odds ratio*	IC95%	p
Sexo (feminino)	2,18	(1,06 – 4,50)	**0,034**	0,85	(0,40–1,77)	0,655
Escolaridade (≤ 9 anos *versus* ≥ 13 anos)	2,35	(1,09–5,08)	**0,030**	4,72	(2,29–9,71)	**< 0,001**
Escolaridade (10–12 anos *versus* ≥ 13 anos)	1,80	(0,96–3,35)	0,065	1,73	(0,93–3,24)	0,084
Uso de álcool na vida (sim)	2,75	(1,31–5,77)	**0,008**	2,47	(1,22–4,98)	**0,011**
Uso de maconha na vida (sim)	5,52	(2,96–10,30)	**< 0,001**	3,66	(1,99–6,73)	**< 0,001**
Uso de cocaína na vida (sim)	2,26	(1,18–4,34)	**0,014**	1,46	(0,74–2,86)	0,275
Uso de crack na vida (sim)	8,61	(2,18–34,07)	**0,002**	4,84	(1,16–20,28)	**0,031**
Crença religiosa (sim)	0,50	(0,27–0,93)	**0,030**	0,82	(0,43–1,57)	0,548
Sinais/sintomas de ansiedade ou depressão (sim)	2,15	(1,26–3,68)	**0,005**	1,15	(0,66–1,98)	0,623

Valores com significância estatística estão apresentados em negrito.

a Área sob a curva ROC = 0,788

b Área sob a curva ROC = 0,735

Como mostrado na Tabela 3, quando comparados aos NF, as variáveis com nível de significância igual ou inferior a 0,05 para os FA foram: ter até 9 anos de escolaridade; não ter emprego; já ter usado álcool, maconha, cocaína e crack; não ter crença religiosa; e apresentar sinais e/ou sintomas de ansiedade ou depressão.

Em relação aos EF, quando comparados aos NF, as variáveis com nível de significância igual ou inferior a 0,05 foram: ter até 9 anos de escolaridade; não ter emprego; estar na classe econômica CDE; ter risco de HIV; uso de álcool, maconha, cocaína e crack na vida; e não ter usado preservativo no último mês (Tabela 3).

Na análise multivariada, as seguintes características foram independentemente associadas ao tabagismo: sexo (feminino); escolaridade (≤ 9 anos); já ter usado álcool, maconha, cocaína e crack; sinais e/ou sintomas de ansiedade ou depressão; e crença religiosa. Maiores chances de ser FA foram encontradas para o sexo feminino (OR = 2,18), pessoas que tinham até 9 anos de escolaridade (OR = 2,35), e pessoas que usaram álcool (OR = 2,75) e drogas ilícitas na vida, tendo sido observada forte associação com o uso de crack (OR = 8,61), maconha (OR = 5,52) e cocaína (OR = 2,26). Foi observada ainda maior chance de ser fumante em pessoas com sinais e/ou sintomas de ansiedade ou depressão (OR = 2,15), e menor chance de ser fumante em pessoas com crença religiosa (OR = 0,50) (Tabela 4).

Na comparação entre EF e NF, as características associadas ao tabagismo foram: ter até 9 anos de escolaridade (OR = 4,72) e já ter usado álcool (OR = 2,47), maconha (OR = 3,66) e crack (OR = 4,84) (Tabela 4).

## DISCUSSÃO

Este estudo revelou elevada prevalência de tabagismo entre PVHIV, sendo o consumo de tabaco entre FA caracterizado por um uso prolongado (acima de 10 anos) e, em sua maior parte, de leve a moderado. O tabagismo atual foi fortemente associado a sexo, escolaridade, uso de substâncias lícitas e ilícitas, e presença de sinais e/ou sintomas de ansiedade ou depressão, enquanto os EF foram associados somente à escolaridade e ao uso de substâncias. Adicionalmente, ter crença religiosa foi fator de proteção contra o tabagismo atual.

As características dos FA neste estudo estão de acordo com os perfis epidemiológicos e dados nacionais e internacionais^3^^–^^8^^,^^15^^,^^16^, sendo essa população composta principalmente por homens, jovens, pessoas com menor nível de escolaridade, com elevado consumo de álcool e drogas ilícitas, e que apresentavam sinais e/ou sintomas de ansiedade ou depressão. O consumo e o tempo de uso do cigarro neste estudo também foram semelhantes ao observado em outras metrópoles no Brasil^5^^,^^6^ e em literatura internacional, ou seja, a maioria dos FA fumavam até 10 cigarros (meio maço) ao dia e por um período acima de 10 anos^4^.

A prevalência de tabagismo encontrada foi de 27,7%, semelhante à reportada em alguns poucos estudos de coorte realizados no país: 28,9% entre 1.815 PVHIV em Recife^5^; 29,9% entre 2.775 PVHIV no Rio de Janeiro^6^, e 33,6% entre 440 PVHIV em um centro de referência em Belo Horizonte^7^. Em contraste, ressalta-se que em coortes internacionais de PVHIV a prevalência de tabagismo é de 40% a 67%^2^^,^^15^^,^^17^.

A menor prevalência de tabagismo no Brasil pode estar relacionada aos efeitos da Política Nacional de Controle do Tabagismo (PNCT), instituída no país desde o final da década de 1980, que levou a uma diminuição geral do tabagismo na população brasileira^8^. Ainda assim, a prevalência de tabagismo atual entre PVHIV encontrada neste estudo é muito superior à da população geral brasileira, estimada em 10,1%^8^. Esse resultado evidencia a necessidade de priorizar intervenções direcionadas a PVHIV com o intuito de cessar o tabagismo.

As PVHIV usuárias de álcool e drogas ilícitas foram mais propensas a ser tabagistas e ex-tabagistas em comparação com os NF, e o uso de crack teve maior associação com o tabagismo. A associação entre álcool, drogas ilícitas e tabagismo em PVHIV tem sido amplamente relatada na literatura nacional e internacional, e essas características estão associadas a piores desfechos clínicos entre PVHIV^3^^–^^7^^,^^18^^,^^19^.

Em outro estudo do Projeto ECOART que acompanhou PVHIV em até 12 meses de TARV, os resultados obtidos a partir de dados de prontuário mostraram que o tabagismo e o uso de drogas ilícitas reduziram a chance de atingir a supressão viral^7^. Como discutido na literatura, a via do citocromo P450 é comum ao metabolismo de nicotina, álcool, drogas ilícitas e medicamentos. Essa via desempenha um papel importante nas interações medicamentosas em PVHIV que usam aquelas substâncias devido à capacidade de indução e inibição enzimática. Ressalta-se que a alta prevalência de dependência e abuso de substâncias lícitas e ilícitas em PVHIV está associada à não adesão à TARV, e consequentemente, à falha na supressão viral^20^.

No Vietnã, uma pesquisa com 409 PVHIV tabagistas mostrou que em torno de 37% e 69% eram usuários de álcool e de drogas, respectivamente, e 41% apresentavam ansiedade^21^.

Em nosso estudo, as PVHIV com sinais e/ou sintomas de ansiedade ou depressão tiveram o dobro de chances de serem fumantes. Esses achados estão de acordo com a coorte do Rio de Janeiro, em que a depressão foi associada de forma independente aos FA em comparação com os NF (OR = 1,38)^6^.

Assim como neste estudo, outros reportaram elevadas taxas de sofrimento mental entre PVHIV (de 25,9% a 66,3%), sendo a depressão e a ansiedade os problemas mais comuns^7^^,^^22^. A presença dessas comorbidades também foi observada em um estudo transversal realizado na China com 360 PVHIV, comparando-se homens e mulheres, em que a prevalência global de sintomas depressivos e ansiosos foi de 66,3% e 45,6%, respectivamente^22^.

Foi mostrado em outro estudo que o distúrbio depressivo frequente e o sofrimento emocional influenciam as altas taxas de tabagismo em PVHIV^23^. Observou-se associação de maior abstinência a partir das medidas de angústia geral, baseadas na ansiedade e na depressão dos estados afetivos aversivos (raiva, ansiedade, depressão, confusão e fadiga), bem como no transtorno de ansiedade generalizada, uma síndrome definida por preocupação excessiva, tensão, fadiga, problemas de concentração e irritabilidade^23^.

A dificuldade de cessar o tabagismo relatada pelos fumantes é que, além de ter de suportar a síndrome de abstinência causada pela dependência física, as outras dificuldades que foram encobertas por esse hábito emergem com toda força, pois os fumantes enxergam o cigarro como um complemento de si mesmos, que fornece alívio, prazer e relaxamento^a^. A cessação do uso de tabaco em PVHIV tende a ser mais difícil, pois diferentes componentes da ansiedade e da depressão estão associados à abstinência dessa substância^23^.

Em nosso estudo, ter crença religiosa foi fator de proteção contra o tabagismo atual. Um estudo com dados do Painel Socioeconômico Alemão (1998–2006) a fim de estimar a relação entre religião e tabagismo concluiu que a religiosidade afeta significativamente o comportamento de fumar, sugerindo que os ateus têm de 13% a 19% mais probabilidade de fumar do que indivíduos religiosos. Segundo os autores, a religião funciona como um canal em que é possível à sociedade impor regras, sendo relevante para os formuladores de políticas de saúde pública considerar a religiosidade ao direcionar intervenções antitabagismo^24^.

Recomenda-se aos programas de cessação de tabagismo o envolvimento de membros da rede social do tabagista devido à sua influência sobre o comportamento relacionado a fumar, com vistas à maior eficácia desses programas^4^.

Quanto às limitações do estudo, não houve verificação bioquímica, considerando-se apenas o relato de uso de cigarros. O viés de desejabilidade social pode ter estado presente, possivelmente levando a subestimar a prevalência de tabagismo por ser este entendido como um comportamento inadequado. Além disso, os dados obtidos a partir dos sistemas de informação Siclom e Siscel poderiam estar incompletos. Como ponto forte, ressalta-se a elevada qualidade da coleta de dados e a robustez do modelo final.

Tornam-se necessárias pesquisas sobre o tabagismo e sua cessação na população PVHIV com vistas a criar uma transição mais rápida da descoberta de riscos para intervenções eficazes.

## CONCLUSÕES

Neste estudo se observou que maior chance de ser FA entre PVHIV foi encontrada para indivíduos que usaram crack, maconha, álcool e/ou cocaína na vida, com menor escolaridade e com sinais e/ou sintomas de ansiedade ou depressão. Para as pessoas com crença religiosa, a chance de ser fumante foi menor.

Este estudo confirma pesquisas anteriores que indicaram que o tabagismo é altamente prevalente entre PVHIV. A prevalência de tabagismo encontrada foi quase o triplo em relação à população geral brasileira, mostrando a necessidade de os serviços de assistência especializada em HIV priorizarem intervenções direcionadas às PVHIV a fim de cessar esse hábito, com abordagem sobre o abuso de álcool e drogas ilícitas, especialmente para pessoas jovens, com baixa escolaridade e com sinais e/ou sintomas de ansiedade ou depressão.

## Footnotes

a Charran IM. Entrevista: Ivone Maria Charran. Entrevista concedida à Ebah. s.l.: Ebah; ago 2018. [citado 31 ago 2018]. Disponível em: http://www.ebah.com.br/content/ABAAABr0sAD/tabagismo
